# Supplementary material for: An open-source framework for large-scale, flexible evaluation of biomedical text mining systems
Source: J Biomed Discov Collab. 2008 Jan 29;3:1. doi: 10.1186/1747-5333-3-1 (PMC2276192; doi:10.1186/1747-5333-3-1)
Supplement: Additional file 1 — Evaluation platform source code. The additional file contains the software evaluation framework discussed in this paper. The most current release can be downloaded from SourceForge.net [1]. See the accompanying README file for instructions on installation and use. Also included in the distribution is a collection of UIMA wrappers for some commonly used BioNLP tools and annotated corpora. [file 1747-5333-3-1-S1.zip › bionlp_uima_repository-1.0.1/docs/api/src-html/edu/uchsc/ccp/util/nlp/parser/corpus/CorpusParser.html]

```
001    package edu.uchsc.ccp.util.nlp.parser.corpus;
002    
003    import java.io.IOException;
004    import java.util.Iterator;
005    
006    import edu.uchsc.ccp.util.nlp.annotation.AnnotationSet;
007    import edu.uchsc.ccp.util.nlp.annotation.Annotator;
008    import edu.uchsc.ccp.util.nlp.annotation.TextAnnotation;
009    import edu.uchsc.ccp.util.nlp.document.GenericDocument;
010    import edu.uchsc.ccp.util.nlp.mention.ClassMention;
011    
012    /**
013     * This is an abstract class for a corpus parser. It follows the <code>Iterator</code> interface, and should return a {@link GenericDocument} object
014     * for each document in the corpus being parsed.
015     * 
016     * @author William A Baumgartner, Jr.
017     * 
018     */
019    public abstract class CorpusParser implements Iterator {
020    
021        /* (non-Javadoc)
022         * @see java.util.Iterator#hasNext()
023         */
024        public abstract boolean hasNext();
025    
026        /* (non-Javadoc)
027         * @see java.util.Iterator#next()
028         */
029        public abstract GenericDocument next();
030    
031        public abstract void close() throws IOException;
032    
033        public void remove() {
034            throw new UnsupportedOperationException("remove() has not been implemented for CorpusParser.");
035        }
036    
037        protected void error(String message) {
038            System.err.println("ERROR -- " + this.getClass().getName().substring(this.getClass().getName().lastIndexOf('.') + 1) + ": "
039                    + message);
040        }
041    
042        protected void warn(String message) {
043            System.err.println("WARN -- " + this.getClass().getName().substring(this.getClass().getName().lastIndexOf('.') + 1) + ": "
044                    + message);
045        }
046    
047        /**
048         * Creates a new TextAnnotation
049         * 
050         * @param documentID
051         * @param documentCollectionID
052         * @param spanStart
053         * @param spanEnd
054         * @param classMentionType
055         * @param coveredText
056         * @param annotator
057         * @param annotationSet
058         * @return
059         */
060        protected static TextAnnotation createTextAnnotation(String documentID, int documentCollectionID, int spanStart, int spanEnd,
061                String classMentionType, String coveredText, Annotator annotator, AnnotationSet annotationSet, int annotationID) {
062            ClassMention cm = new ClassMention(classMentionType);
063            TextAnnotation ta = new TextAnnotation(spanStart, spanEnd, coveredText, annotator, annotationSet, annotationID,
064                    documentCollectionID, documentID, -1, cm);
065    
066            return ta;
067        }
068    
069    }
```
